# Temporal Downscaling of Crop Coefficient and Crop Water Requirement from Growing Stage to Substage Scales

**DOI:** 10.1100/2012/105487

**Published:** 2012-04-24

**Authors:** Songhao Shang

**Affiliations:** State Key Laboratory of Hydroscience and Engineering, Department of Hydraulic Engineering, Tsinghua University, Beijing 100084, China

## Abstract

Crop water requirement is essential for agricultural water management, which is usually available for crop growing stages. However, crop water requirement values of monthly or weekly scales are more useful for water management. A method was proposed to downscale crop coefficient and water requirement from growing stage to substage scales, which is based on the interpolation of accumulated crop and reference evapotranspiration calculated from their values in growing stages. The proposed method was compared with two straightforward methods, that is, direct interpolation of crop evapotranspiration and crop coefficient by assuming that stage average values occurred in the middle of the stage. These methods were tested with a simulated daily crop evapotranspiration series. Results indicate that the proposed method is more reliable, showing that the downscaled crop evapotranspiration series is very close to the simulated ones.

## 1. Introduction

Crop water requirement, the essential data for agricultural water management and regional water resources planning, is defined as the amount of water needed to compensate the water consumed by plant transpiration and soil evaporation from cropped field under nonrestricting soil conditions [1]. The values of crop water requirement in any growing periods are equal to crop evapotranspiration (ET_*c*_) under nonrestricting soil conditions in the same period. Therefore, crop water requirement was usually measured or calculated through ET_*c*_ [2].

In 1998, Food and Agriculture Organization (FAO) recommended an updated procedure to calculate ET_*c*_ from reference evapotranspiration (ET_0_) and crop coefficient (*K*_*c*_) [2]. ET_0_ is mainly influenced by meteorological factors and can be calculated by the Penman-Monteith equation, and *K*_*c*_  is mainly influenced by the crop type, its growing stage, and climate factors. Allen et al. [2] used a piecewise line to depict the variation of *K*_*c*_ with crop development and gave tabulated values of *K*_*c*_ in the initial and midseason stages and at the end of the late growing stage for main crops. However, this generalized crop coefficient curve and these tabulated values of *K*_*c*_ represent general crop conditions and cannot fully consider differences of crop varieties and local environment.

On the other hand, crop coefficient and crop water requirement had been studied for main crops in many places over the world. For example, an irrigation experiment network with over 100 stations had studied crop water requirement in China during the past several decades, and results of crop coefficient and crop water requirement for main crops were available at the temporal scale of growing stage [3]. These results can be spatially interpolated to obtain crop coefficient and crop water requirement of growing stages in unmeasured sites. However, in agricultural water management and regional water resources planning, crop water requirement in different time scales is usually necessary, such as monthly, weekly, or even daily scales. Therefore, it is necessary to downscale or disaggregate crop water requirement from growing stages to values in shorter periods.

Since crop evapotranspiration is usually measured over specified growing periods, it belongs to flow variables [4]. Temporal disaggregation methods for flow variables have been widely used in many disciplines, such as hydrology [5, 6], meteorology [7, 8], and economics [9, 10]. Main temporal disaggregation methods include pure mathematical methods without auxiliary information [11], time series analysis models [5, 12], regression models [13], and dynamic models [14]. *Eurostat* [5], *Feijoó *et al. [9] and *Bojilova* [10] compared main methods for temporal disaggregation. However, these methods usually require a large number of data [12] or depend on assumptions on the smoothness of the flow variables and/or some plausible minimizing criteria [11].

In this paper, we proposed a method to downscale crop coefficient and crop water requirement from growing stage to substage scales using crop evapotranspiration data in growing stages and reference evapotranspiration data in both stages and substages. This method was compared with two direct interpolation methods and tested with a simulated crop evapotranspiration series of winter wheat at Xiaohe Station in North China.

## 2. Method to Temporally Downscale Crop Coefficient and Crop Water Requirement

For flow variables whose values are measured over specified periods, interpolation methods are not applicable directly to estimate their values over unknown periods or points. To use direct interpolation method, one straightforward method is to assume that stage average values occurred in the middle of the stages [7]. However, this assumption is valid for linear case and usually invalid for nonlinear cases. For crop evapotranspiration, direct interpolation of crop evapotranspiration or crop coefficient can be used following the above linear assumption.

**Figure 1:**
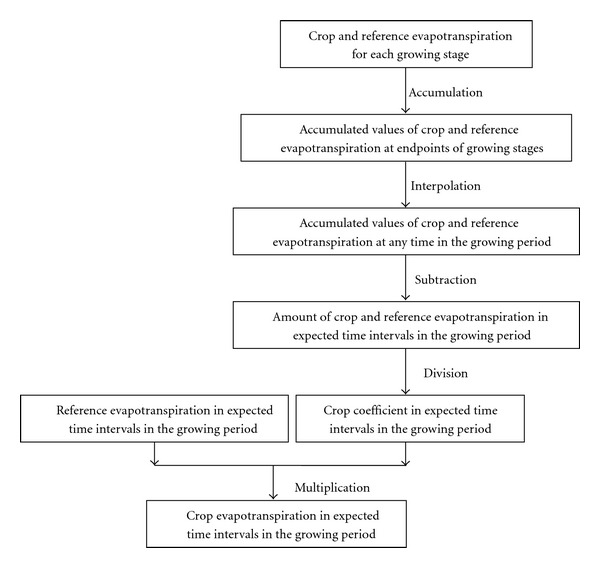
Proposed procedure to temporally downscale crop coefficient and crop water requirement.

To avoid the unrealistic linear assumption, an alternative method to temporally downscale crop coefficient and crop water requirement was proposed, which was based on the interpolation of accumulated crop and reference evapotranspiration. This method used the interpolation of accumulated evapotranspiration rather than evapotranspiration itself. Considering that the accumulated crop evapotranspiration is a stock variable, it can be used for direct interpolation to approximate accumulated crop evapotranspiration at any time of the growing period. Then values of crop evapotranspiration in any expected periods can be estimated. Considering the match of crop and reference evapotranspiration in calculating the crop coefficient, reference evapotranspiration at growing stage scale is also disaggregated similarly. Procedure of this method is shown in Figure 1.

Suppose that the whole or part crop growing period is divided into *n* stages, the duration of stage *i*  (*i* = 1, 2,…, *n*) is Δ*t*_*i*_, and measured or simulated crop evapotranspiration (crop water requirement) during stage *i* is ET_*ci*_. Reference evapotranspiration in both growing stages (ET_0*i*_) and substages can be calculated by the Penman-Monteith equation [2] with monitored meteorological data, which is used as an auxiliary variable to downscale crop coefficient and crop evapotranspiration. The accumulated crop and reference evapotranspiration at the beginning and end of growing stages can be calculated from crop and reference evapotranspiration in growing stages with (1) and (2), respectively:

(1) $$\begin{array}{c} \text{AE}{\text{T}}_{c}\left(t_{0}\right)=0,\\ \text{AE}{\text{T}}_{c}\left(t_{i}\right)=\overset{i}{\underset{k=1}{\sum }}\text{E}{\text{T}}_{ck}=\text{AE}{\text{T}}_{c}\left(t_{i-1}\right)+\text{E}{\text{T}}_{\text{c}i},\;i=1,\;2,\ldots ,\;n, \end{array}$$

(2) $$\begin{array}{c} \text{AE}{\text{T}}_{0}\left(t_{0}\right)=0,\\ \text{AE}{\text{T}}_{0}\left(t_{i}\right)=\overset{i}{\underset{k=1}{\sum }}\text{E}{\text{T}}_{0k}=\text{AE}{\text{T}}_{0}\left(t_{i-1}\right)+\text{E}{\text{T}}_{0i},\;\;i=1,\;2,\ldots ,\;n, \end{array}$$

where AET*_c_*(*t*_*i*_) and AET_0_(*t*_*i*_) are accumulated crop and reference evapotranspiration at time *t*_*i*_, and *t*_*i*_ is defined as

(3) $$\begin{array}{c} t_{0}=0,\;\;t_{i}=\overset{i}{\underset{k=1}{\sum }}\Delta t_{k}=t_{i-1}+\Delta t_{i},\;i=1,\;2,\ldots ,n.\; \end{array}$$

Interpolation polynomials or piecewise polynomials [15], *S*_*c*_(*t*) and *S*_0_(*t*), can be constructed to approximate accumulated crop and reference evapotranspiration using interpolation conditions of (1) and (2), respectively. When choosing appropriate type of interpolation polynomials or piecewise polynomials, the number of interpolation nodes (*n* + 1) and the characters of the accumulated evapotranspiration should be considered. In general, polynomials are appropriate for smaller *n*, and piecewise polynomials for larger *n*. Moreover, interpolation functions for accumulated crop and reference evapotranspiration should be strictly increasing functions since values for crop and reference evapotranspiration are always positive. Therefore, the monotone piecewise cubic interpolation method proposed by Fritsch and Carlson (1980) [16] is appropriate for larger *n*.

From *S*_*c*_(*t*) and *S*_0_(*t*), the amount of crop and reference evapotranspiration over expected time interval [*t*_*u*_,*t*_*v*_] (0 ≤ *t*_*u*_ < *t*_*v*_ ≤ *t*_*n*_) in the growing period can be estimated with (4) and (5), respectively:

(4) $$\text{E}{\text{T}}_{c}^{i}\left(t_{u},t_{v}\right)=S_{c}\left(t_{v}\right)-S_{c}\left(t_{u}\right),\;0\leq t_{u}<t_{v}\leq t_{n},$$

(5) $$\text{E}{\text{T}}_{0}^{i}\left(t_{u},t_{v}\right)=S_{0}\left(t_{v}\right)-S_{0}\left(t_{u}\right),\;0\leq t_{u}<t_{v}\leq t_{n},$$

where superscript *i* refers to interpolated values. Specially, daily crop and reference evapotranspiration in the day of *t*_*u*_ (*t*_*u*_ = 1,2,…, *n*) can be estimated with (6) and (7), respectively:

(6) $$\text{E}{\text{T}}_{c}^{i}\left(t_{u}\right)=S_{c}\left(t_{u}\right)-S_{c}\left(t_{u}-1\right),\;t_{u}\;=1,\;2,\ldots ,\;n,$$

(7) $$\text{E}{\text{T}}_{0}^{i}\left(t_{u}\right)=S_{0}\left(t_{u}\right)-S_{0}\left(t_{u}-1\right),\;t_{u}\;=1,\;2,\ldots ,\;n.$$

Since only crop and reference evapotranspiration in limited growing stages were available for interpolation, fluctuations of evapotranspiration in higher frequencies were smoothed out. Therefore, the disaggregated evapotranspiration varies smoothly over time, which represents the trend of evapotranspiration processes.

Using interpolated crop and reference evapotranspiration in substage periods, crop coefficient can be calculated as the ratio of crop to reference evapotranspiration, that is,

(8) $$K_{c}^{d}=\frac{\text{E}{\text{T}}_{c}^{i}}{\text{E}{\text{T}}_{0}^{i}},$$

where *K*_*c*_^*d*^ is the downscaled crop coefficient in a substage period, and ET_*c*_^*i*^ and ET_0_^*i*^ are interpolated crop and reference evapotranspiration in the same substage period.

Owing to the smoothness of disaggregated crop and reference evapotranspiration, the variation of downscaled crop coefficient is also smooth over time. To reflect the fluctuation of crop evapotranspiration over growing period, downscaled crop evapotranspiration (ET_*c*_^*d*^) can be calculated with the product of actual reference evapotranspiration and the downscaled crop coefficient with (9):

(9) $$\text{E}{\text{T}}_{c}^{d}=K_{c}^{d}\text{E}{\text{T}}_{0}.$$

## 3. Data and Evaluation Criteria for the Downscaling Method

To evaluate the effectiveness of the proposed method to temporally downscale crop coefficient and crop water requirement, a daily crop evapotranspiration series without soil water stress to crop simulated with a soil water balance model [17] was used. This series includes 110 values of daily crop evapotranspiration of winter wheat from March 6 (greening) to June 23 (harvesting) in 2003 at Xiaohe Experiment Station in Shanxi province in North China.

These 110 values were aggregated to 4 values of crop evapotranspiration in four stages (Table 1), which were then downscaled to daily scale to evaluate the performance of different disaggregation methods.

Besides the proposed method, direct interpolation of crop evapotranspiration and crop coefficient by assuming that stage average values occurred in the middle of the stage were also used for comparison (Table 2).

When actual values of crop evapotranspiration in substage periods are available from measurement or simulation, the scatter plot of original (ET_*c*_^o^) versus downscaled (ET_*c*_^*d*^) crop evapotranspiration can be used to evaluate the performance of the downscaling method through visual inspection and regression analysis. If most of the scatter points are close to the 1 : 1 line, then the agreement between original and downscaled crop evapotranspiration is more likely to be good. The linear regression equation between ET_*c*_^o^ and ET_*c*_^*d*^ can be expressed as

(10) $$\text{E}{\text{T}}_{c}^{d}=a+b\text{E}{\text{T}}_{c}^{\text{o}}+e,$$

where *a* and *b* are the intercept and slope of the regression line, respectively, and *e* is the residual. The slope *b*, the intercept *a*, and the Pearson product moment correlation coefficient *r* can be calculated from

(11) $$b=\frac{\sum_{j=1}^{N}\left(\text{E}{{\text{T}}_{c,}^{\text{o}}}_{j}-\bar{\text{E}{\text{T}}_{c}^{\text{o}}}\right)\left(\text{E}{{\text{T}}_{c,}^{\text{d}}}_{j}-\bar{\text{E}{\text{T}}_{c}^{d}}\right)}{\sum_{j=1}^{N}{\left(\text{E}{{\text{T}}_{c,}^{\text{o}}}_{\text{j}}-\bar{\text{E}{\text{T}}_{c}^{\text{o}}}\right)}^{2}},$$

(12) $$a=\bar{\text{E}{\text{T}}_{c}^{d}}-b\bar{\text{E}{\text{T}}_{c}^{\text{o}}},$$

(13) $$r=\frac{\sum_{j=1}^{N}\left(\text{E}{{\text{T}}_{c,}^{\text{o}}}_{\text{j}}-\bar{\text{E}{\text{T}}_{c}^{\text{o}}}\right)\left(\text{E}{{\text{T}}_{c,}^{d}}_{\text{j}}-\bar{\text{E}{\text{T}}_{c}^{d}}\right)}{{\left[\sum_{j=1}^{N}{\left(\text{E}{{\text{T}}_{c,j}^{\text{o}}}_{}-\bar{\text{E}{\text{T}}_{c}^{\text{o}}}\right)}^{2}\sum_{j=1}^{N}{\left(\text{E}{{\text{T}}_{c,j}^{d}}_{}-\bar{\text{E}{\text{T}}_{c}^{d}}\right)}^{2}\right]}^{1/2}},$$

where *N* is the number of data points, $\text{and}\;\bar{\text{E}{\text{T}}_{c}^{\text{o}}}$ and $\bar{\text{E}{\text{T}}_{c}^{d}}$ are average values of the original and downscaled crop evapotranspiration, respectively. For perfect downscaling results, *a* would be 0, and *b* and *r* would be 1.

Relative volume error (RVE) and root mean squared error (RMSE) are also two widely used indexes for model evaluation. RVE represents the volume conservative characteristic of a disaggregation method, while RMSE represents the level of overall agreement between the original and downscaled crop evapotranspiration. They are defined as

(14) $$\text{RVE}=\frac{\sum_{j=1}^{N}\left(\text{E}{\text{T}}_{c,\text{j}}^{\text{o}}-\text{E}{\text{T}}_{c,\text{j}}^{d}\right)}{\sum_{j=1}^{N}\text{E}{\text{T}}_{c,j}^{o}},$$

(15) $$\text{RMSE}={\left[\frac{1}{N}\overset{N}{\underset{j=1}{\sum }}{\left(\text{E}{\text{T}}_{c,j}^{d}-\text{E}{\text{T}}_{c,j}^{\text{o}}\right)}^{2}\right].}^{1/2}$$

Both RVE and RMSE would be 0 for perfect downscaling results.

## 4. Results and Discussions

### 4.1. Crop Coefficient

Crop coefficients in substage periods can be estimated with methods 2 and 3 in Table 2.

**Figure 2:**
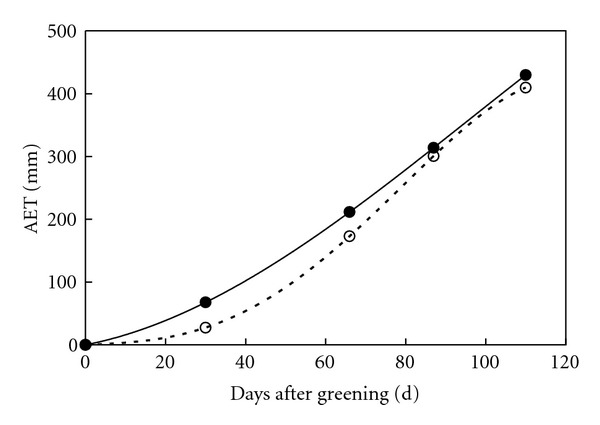
Accumulated values of reference (dot) and crop (circle) evapotranspiration and corresponding interpolation polynomials (solid and dashed lines) from greening to harvesting of winter wheat in 2003.

For method 3, the accumulated values of crop and reference evapotranspiration were calculated from their values in four growing stages and used to construct interpolation polynomials. Considering that the numbers of accumulated values were five, quartic polynomials with constants equaling to 0 were used. Interpolation polynomials (Figure 2) for accumulated crop and reference evapotranspiration were (16) and (17), respectively:

(16) $$\text{AE}{\text{T}}_{c}^{i}=-918.135t^{\prime^{4}}+1346.967t^{\prime^{3}}-52.342t^{\prime^{2}}+33.099t^{\prime },$$

(17) $$\text{AE}{\text{T}}_{0}^{i}=-24.764t^{\prime^{4}}-127.654t^{\prime^{3}}+445.714t^{\prime^{2}}+136.390t,$$

where *t*′ = *t*/110 is the relative time, and *t* is days after greening. These two interpolation polynomials are appropriate since they are both strictly increasing functions.

**Figure 3:**
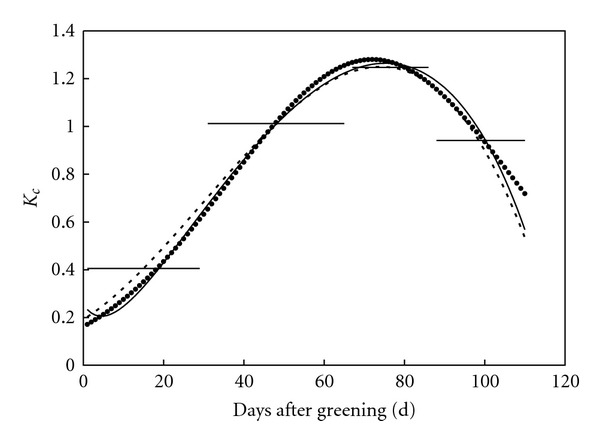
Daily crop coefficient (dot), average ones at different growing stages (thick solid line), and disaggregated ones with methods 2 (dashed line) and 3 (solid line).

From Figure 2, reference evapotranspiration tends to increase from greening (early March) to harvesting (late June), while crop evapotranspiration increases slowly in greening and milking stages and quickly in jointing and heading stages. Consequently, the increase of accumulated reference evapotranspiration is superlinear, while the variation of accumulated crop evapotranspiration follows a sigmoid pattern. The interpolation polynomials in Figure 2 were used to calculate reference and crop evapotranspiration in any time intervals during the crop growing period using (4) and (5). Then crop coefficients in these intervals were calculated with (8), and downscaled daily crop coefficient is shown in Figure 3.

For method 2, crop coefficients in the middle of growing stages were assumed to be the stage average values, and crop coefficients in substage periods were estimated with the corresponding interpolation polynomial (Figure 3).

Disaggregated daily crop coefficients were compared with the original and stage average values (Figure 3). In early growing periods, method 2 tends to overestimate the crop coefficient. In final growing periods, both methods 2 and 3 underestimate the crop coefficient. In general, disaggregated daily crop coefficients are both acceptable compared with the original ones, and the results of method 3 are slightly superior to those of method 2.

### 4.2. Crop Evapotranspiration

**Figure 4:**
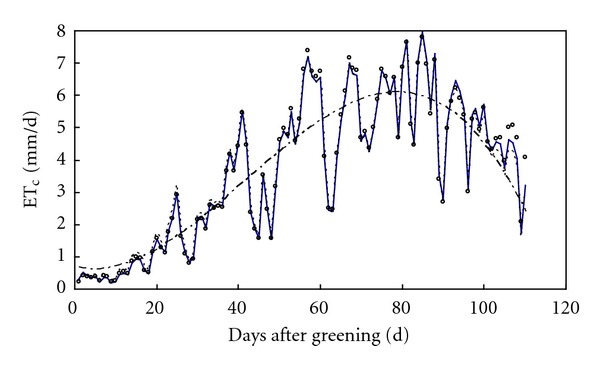
Original daily crop evapotranspiration (circle) and disaggregated ones with methods 1 (dash-dotted line), 2 (dashed line), and 3 (solid line).

Crop evapotranspiration can be disaggregated with all three methods in Table 2.  Figure 4 shows the variations of original and disaggregated crop evapotranspiration on a daily basis. Disaggregated results of methods 1 vary smoothly over time as expected, which represents the smoothed trend of original daily evapotranspiration. On the other hand, disaggregated results of methods 2 and 3 are very close. Moreover, their fluctuations are very similar to original simulated ones, since daily reference evapotranspiration is used in these two disaggregation methods.

Values of evaluation criteria for these three disaggregation methods are listed in Table 3. Interpolation of crop evapotranspiration with the linear assumption (Method 1) gave the worst results for all evaluation criteria except REV, while interpolation of accumulated crop and reference evapotranspiration (method 3) gave the best results. Therefore, method 3 is the most appropriate method to disaggregate crop coefficient and crop evapotranspiration from stage to substage scales. Interpolation of crop coefficient with the linear assumption (Method 2) is also acceptable.

## 5. Conclusions

We proposed a method to estimate crop coefficient and crop water requirement in substage scales from crop evapotranspiration in growing stages and reference evapotranspiration in growing stages and substages. In this method, accumulated values of crop and reference evapotranspiration calculated from their values in growing stages were used to estimate crop and reference evapotranspiration in substage periods through interpolation, which were then used to obtain the crop coefficient in substage periods. The crop water requirement in substage periods was estimated with downscaled crop coefficient and the reference evapotranspiration at substage scale.

The method was tested with a simulated daily crop evapotranspiration series. Results indicate that the variation of crop coefficient is close to the simulated one, and the downscaled crop evapotranspiration series is very close to the original one and superior to two direct interpolation methods.

Since crop coefficient and crop water requirement in growing stages for main crops are available in many irrigation experiment stations [3], they can be used to obtain crop coefficient and crop water requirement in shorter time interval using the present downscaling method. The downscaled values are more useful in agricultural water management and regional water resources planning.

## Figures and Tables

**Table 1:** Values of reference (*ET*_0_) and crop (*ET*_*c*_) evapotranspiration and crop coefficient (*K*_*c*_) in growing stages of winter wheat after greening in 2003.

Growing stage	Starting date	Ending date	Duration (*d*)	ET_0_ (mm)	ET_*c*_ (mm)	*K*_*c*_
Greening	March 6	April 4	30	67.6	27.4	0.40
Jointing	April 5	May 10	36	143.9	145.6	1.01
Heading	May 11	May 31	21	102.3	127.6	1.25
Milking	June 1	June 23	23	115.8	109.0	0.94
Greening to harvest	March 6	June 23	110	429.6	409.6	0.95

**Table 2:** Disaggregation methods for crop coefficient (*K*_*c*_) and crop evapotranspiration (*ET*_*c*_).

No.	Disaggregation method	Input	Output
1	Interpolation of crop evapotranspiration with the linear assumption	ET_*c*_ in growing stages	ET_*c*_ in substages
2	Interpolation of crop coefficient with the linear assumption	*K*_*c*_ in growing stages and ET_0_ in substages	ET_*c*_ and *K*_*c*_ in substages
3	Interpolation of accumulated crop and reference evapotranspiration	ET_*c*_ and ET_0_ in growing stages and ET_0_ in substages	ET_*c*_ and *K*_*c*_ in substages

**Table 3:** Evaluation of different disaggregation methods.

Method	Total ET_*c*_ (mm)	REV	RMSE (mm/d)	*a*	*b*	*r*
1	406.5	0.0075	1.12	0.939	0.740	0.865
2	404.3	0.0129	0.22	0.112	0.957	0.996
3	406.8	0.0068	0.16	−0.010	0.996	0.998

Perfect value	409.6	0	0	0	1	1
